# Capturing adolescents in need of psychiatric care with psychopathological symptoms: A population-based cohort study

**DOI:** 10.1192/j.eurpsy.2021.2251

**Published:** 2021-11-29

**Authors:** Anat Rotstein, Judy Goldenberg, Suzan Fund, Stephen Z. Levine, Abraham Reichenberg

**Affiliations:** 1 Department of Psychiatry, Icahn School of Medicine at Mount Sinai, New York, New York, USA; 2 Department of Behavioral Sciences, Israel Defense Forces, Tel Aviv, Israel; 3 Department of Community Mental Health, University of Haifa, Haifa, Israel; 4 The Department of Environmental Medicine and Public Health, Icahn School of Medicine at Mount Sinai, New York, New York, USA; 5 The Mindich Child Health and Development Institute, Icahn School of Medicine, New York, New York, USA

**Keywords:** Classification, clinical prediction, detection and prevention, mental disorders in adolescence, referral to a mental health professional, the Brief Symptom Inventory

## Abstract

**Background:**

The current study aims to overcome past methodological limitations and capture adolescents in need of psychiatric care with psychopathological symptoms in a cohort with unrestricted access to mental health professionals.

**Methods:**

The study source population consisted of a random sample of adolescents aged 16-17 years (N=1,369) assessed by the Israeli Draft Board. An adapted version of the Brief Symptom Inventory was used to identify clinically relevant psychopathological symptoms with scores categorized as severe if they were in the top 10th percentile of symptoms, otherwise not severe. An independent interview with a subsequent referral to a mental health professional was used to categorize adolescents in need of psychiatric care. To examine the association between severe psychopathological symptoms and the need for psychiatric care, logistic regression models were fitted unadjusted and adjusted for age, sex, and intellectual assessment scores. Adjusted classification measures were estimated to examine the utility of severe psychopathological symptoms for clinical prediction of need for psychiatric care.

**Results:**

Information on 1,283 adolescents was available in the final analytic sample. Logistic regression modeling showed a statistically significant (p<0.001) association between self-reported severe psychopathological symptoms and the need for psychiatric care (OR adjusted: 4.38; 95% CI: 3.55–5.40). Severe psychopathological symptoms had a classification accuracy of 83% (CI: 81%–85%).

**Conclusions:**

Severe psychopathological symptoms, although accounting for a fair proportion of treatment seeking, would perhaps be better useful for classification purposes alongside other variables rather than in isolation.

## Introduction

The transition from adolescence to adulthood is one of the most influential developmental stages across the lifespan [1]. Adolescence is a critical period for neurodevelopment, when over half of all lifetime psychiatric disorders begin [2, 3]. Mental disorders account for approximately 45% of the global disease burden in adolescents [4], and are associated with multiple developmental concerns (e.g., lower educational achievement; [5]). Research on adolescents suggests that contact with mental health professionals has preventive effects against psychiatric disorders [6, 7] and is cost-effective [8]. However, surveys estimate that approximately 67% of adolescents needing services, as defined by the presence of a psychiatric disorder, neither seek nor receive formal help [9].

Widespread methodological limitations in the literature appear to obscure the avenues to investigate prevention strategies for adolescents at-risk. First, most existing research is based on restricted care access due to financial and regional barriers [10]. Financial resources for adolescent mental health care are insufficient and mental health services for adolescents are most likely less than are needed [11]. A major challenge in this area is the shortage of mental health professionals [5]. Furthermore, mental hospitals, which are the main axis of mental health care, are found in major cities only [12] thus forming regional barriers. To date, no study has examined unrestricted mental health care access in adolescents. Unrestricted access to mental health care is based on the notion that mental health care should be accessible to all persons at all times and locations [13]. Second, most studies include informal mental health resources rather than focusing on specialized mental health professionals [14]. Informal mental health resources include all nonprofessional sources available in the community (e.g., friends; [14]). Although informal mental health resources form a broader measure of mental health care, they may not provide effective treatment [14]. Third, no study has examined clinically relevant classification indices. Classification indices quantify the extent a binary exposure (e.g., surpassing a symptom threshold) overlaps with a binary outcome (e.g., the need for psychiatric care). The classification model is usually presented through a standard two by two tables (Table 1; [15]) and its attributes can be measured based on several indices (Table 2; [15]). Classification indices facilitate the quantification of the performance of different measures and therefore play an influential role in the assessment of diagnostic effectiveness. These indices form the basis for the decision of whether to implement early detection and prevention measures in clinical practice [16].

**Table 1.** Classification model: severe psychopathological symptoms and need of psychiatric care.

Outcome	Categorized as in need of psychiatric care	Categorized as not in need of psychiatric care
Exposure		
Categorized as with severe psychopathological symptoms	True positive	False negative
Categorized as without severe psychopathological symptoms	False positive	True negative

**Table 2.** Description of the classification indices.

Classification index	Definition	Concrete example based on severe psychopathological symptoms and a need for psychiatric care	Formula
True positive	The *number* of true positives cases correctly classified as such	The number of participants classified as with severe psychopathological symptoms who were also classified as in need of psychiatric care	
False positive	The *number* of true negative cases misclassified as positives	The number of participants classified as without severe psychopathological symptoms who were also classified as in need of psychiatric care	
True negative	The *number* of true negative cases correctly classified as such	The number of participants classified as without severe psychopathological symptoms who were not classified as in need of psychiatric care	
False negative	The *number* of true positive cases misclassified as negatives	The number of participants classified as with severe psychopathological symptoms who were not classified as in need of psychiatric care	
Sensitivity	Of all true positive cases, the *proportion* of those correctly classified as such	Of all participants classified as with severe psychopathological symptoms, the proportion of participants who were classified as in need of psychiatric care	(TP)/(TP + FN)
Specificity	Of all true negative cases, the *proportion* of those correctly classified as such	Of all participants not classified as with severe psychopathological symptoms, the proportion of participants who were not classified as in need of psychiatric care	(TN)/(TN + FP)
Accuracy	Of all cases, the *proportion* of correctly classified cases, both positive and negative	Of all participants, the combined proportion of participants classified as with severe psychopathological symptoms who were classified as in need of psychiatric care and those classified without severe psychopathological symptoms who were not classified as in need of psychiatric care	(TN + TP) / (TN + FP + TP + FN)
Positive predictive value	The *probability* that true positives are classified as such	The probability that participants classified as with severe psychopathological symptoms will be classified as in need of psychiatric care	(TP)/(TP + FP)
Negative predictive value	The *probability* that true negatives are classified as such	The probability that participants classified as without severe psychopathological symptoms will not be classified as in need of psychiatric care	(TN)/(TN + FN)
Number needed to diagnose	The number of cases that need to be classified as positive to detect one true positive correctly	The number of participants who need to be classified as in need of psychiatric care to correctly detect one participant with severe psychopathological symptoms in the total study population	1/(sensitivity + specificity − 1)

Abbreviations: FN, false negative; FP, false positive; NND, number needed to diagnose; NPV, negative predictive values; PPV, positive predictive values; TN, true negative; TP, true positive.

The current study aims to capture adolescents in need of psychiatric care with psychopathological symptoms, focusing on clinically relevant classification measures. This study is based on an adolescent cohort in a setting without any health access inequalities, psychometric assessments of psychopathological symptoms, and external referrals to mental health professionals.

## Methods

The Institutional Review Board at the University of Haifa granted ethical approval to conduct the study with a waiver of informed consent (Application no. 090/21).

### Study population and procedure

Adolescents in Israel undergo a mandatory predraft screening by the Israeli Draft Board at age 16–17 years to ascertain their eligibility to serve in the military. This assessment includes individuals who are eligible for military service, as well as those who will be excused from service based on medical, psychiatric, or social grounds. The study sample (*N* = 1,421), provided by the Israeli Draft Board, included any individual coming in for a standard mandatory screening on randomly selected days in 2017, with a minimal Hebrew language proficiency level. For the analytic sample, we excluded individuals older than 17 (*N* = 52) leaving a sample of 1,369 (average age of 16.96; SD = 0.22). See Supplementary Figure S1 for a flow diagram. Participants were administered a self-report psychopathological symptoms questionnaire, a computerized intellectual assessment, and a behavioral screening interview. Based on the screening interview, adolescents with a suspected psychiatric disorder were referred to a mental health professional and defined as in need of psychiatric care. Data were retrieved from computerized military files.

### Psychopathological symptoms

The Brief Symptom Inventory (BSI; [17]) is a self-report measure used to identify clinically relevant psychopathological symptoms in adolescents and adults. It consists of 53 items covering nine symptom dimensions: somatization, obsession–compulsion, interpersonal sensitivity, depression, anxiety, hostility, phobic anxiety, paranoid ideation, and psychoticism. Ratings characterize the intensity of distress during the past month. An adapted version of the BSI was supplemented with five items to cover two more dimensions: drug use and self-harming behaviors. All items were responded to on a 5-point scale ranging from 0 (not at all) to 4 (extremely). The measure took approximately 10–12 min to complete. The internal reliability reported for this measure, based on a previous sample of adolescents, was satisfactory (alpha = 0.95 for the general severity index; average alpha = 0.71 for all BSI subscales) as was the convergence validity with the General Well-Being questionnaire [18] (*r* = −0.62 for the general severity index; average *r* = −0.49 for all BSI subscales) [19].

### Intellectual assessment

The Israeli Draft Board intellectual assessment includes four cognitive tests that measure verbal understanding and abstraction, categorization abilities, mathematical reasoning, and visual–spatial problem-solving abilities. The summary score of the cognitive test battery has been found to be a highly valid measure of general intelligence as measured by the Wechsler Adult Intelligence Scale [20] total score (*r* > 0.90). The results of the intellectual assessment were further significantly correlated with external measures (i.e., rank upon discharge; *r* > 0.41) [21]. This intellectual assessment has been used in many other studies [22–25].

### Need for psychiatric care

An interview assessing personality and behavioral traits was administered by college-aged individuals who participated in a four-month-long training course on the administration of the interview These administrators, affiliated with the Behavioral Science Division, are routinely overseen by professional mental health specialists [26]. Based on the interview and on findings from a general physician’s examination, adolescents who were suspected of having behavioral disturbances or mental illnesses were referred to an in-depth assessment by a mental health professional (a clinical social worker or psychologist, affiliated with the Mental Health Division). Criteria for referral to an in-depth mental health assessment include a history of psychological or psychiatric treatment or complaints, manifestation of behavioral abnormalities during the physician’s examination or the screening interview, or obtaining the lowest score on the rating of social functioning in the screening interview [27]. The test–retest reliability of the screening interview, made after several days by different interviewers, was high (>0.8) as was its validity in predicting external measures (i.e., rank after 30 months of military service; *r* = 0.39) [21, 26].

### Data analyses

First, data were screened for missing values and completeness. Second, the primary analysis was conducted. Previous research has found that the percentages of patients attaining levels of high distress on the nine BSI scales are up to 10% (highest being 9.5%) [28]. Therefore, BSI scores were categorized as severe if they were in the top 10th percentile of symptoms, otherwise not severe. Logistic regression models were fitted to quantify the association between severe psychopathological symptoms and the need for psychiatric care with odds ratios (OR) and their associated 95% confidence intervals (CI). Logistic regression models were computed for total BSI scores and for each of the eleven subscales (because certain disorders are stronger indicators of a need for care; [29]) unadjusted (without covariates) and adjusted for covariates (age, sex, and the total intellectual assessment score). Next, the utility of severe psychopathological symptoms for clinical prediction of need for psychiatric care was ascertained based on classification indices of each adjusted logistic regression model (Tables 1 and 2). There are no clear guidelines regarding sufficient sensitivity and specificity [30], although values of 90% and over may be considered to be sufficiently reliable as to have public health policy implications.

Third, to test the robustness of the primary analysis, sensitivity analyses were conducted, restricted to relevant subgroups of participants: participants with a low intellectual assessment score, defined as lower than two standard deviations under the population mean (because lower intellectual ability is related to severe mental disorders; [22, 31]); as well as males and females (because a previous Israeli study based on a nationwide representative sample has shown that females tend to report higher levels of symptoms than males; [32]). Each sensitivity analysis repeated the primary analysis, except the covariate that the analysis was restricted to was dropped. Finally, to ensure the results were not an artifact of the use of 10% as a symptom severity categorization threshold, we reanalyzed the data by altering the BSI symptomatic threshold to 20% (because previous research has found that the percentages of patients attaining levels of moderate distress on the nine BSI scales average to about 20%; *X* = 19.75) [28]. All analyses were computed in R version 4.1.0 [33].

## Results

### Sample characteristics

Individuals with missing data on sex (*N* = 67), intellectual assessment (*N* = 18) and psychopathological symptoms (*N* = 1) were excluded (6.28% missing in total; *N* = 86) leaving a total of 1,283 adolescents for analysis. Characteristics of the analytic sample show statistically significant (*p* < 0.05) differences between adolescents in need of psychiatric care and those who are not (Table 3).

**Table 3.** Sample characteristics.

	Total sample	Categorized as not in need of psychiatric care	Categorized as in need of psychiatric care	*p*-value
*N*	1,283	976	307	
Age: Mean (SD)	16.96 (0.22)	16.96 (0.21)	16.98 (0.24)	0.071
Birth year: Mean (SD)	2000.46 (0.50)	2000.48 (0.50)	2000.41 (0.49)	0.034
Intellectual assessment score: Mean (SD)	56.96 (18.32)	58.90 (17.90)	50.78 (18.29)	<0.001
Somatization: Mean (SD)	1.69 (3.06)	0.96 (1.94)	4.00 (4.49)	<0.001
Obsession–compulsion: Mean (SD)	2.75 (3.38)	1.74 (2.15)	5.97 (4.43)	<0.001
Interpersonal sensitivity: Mean (SD)	1.31 (2.33)	0.75 (1.40)	3.09 (3.49)	<0.001
Depression: Mean (SD)	2.32 (3.41)	1.38 (1.97)	5.29 (4.96)	<0.001
Anxiety: Mean (SD)	2.78 (3.45)	1.82 (2.12)	5.80 (4.86)	<0.001
Hostility: Mean (SD)	1.71 (2.71)	1.00 (1.50)	3.98 (4.11)	<0.001
Phobic anxiety: Mean (SD)	0.91 (2.21)	0.40 (1.07)	2.50 (3.67)	<0.001
Paranoid ideation: Mean (SD)	2.29 (3.18)	1.38 (2.05)	5.18 (4.23)	<0.001
Psychoticism: Mean (SD)	1.23 (2.38)	0.54 (1.08)	3.43 (3.70)	<0.001
Drug use: Mean (SD)	0.33 (0.77)	0.23 (0.51)	0.65 (1.24)	<0.001
Self-harming behaviors: Mean (SD)	0.08 (0.27)	0.00 (0.03)	0.32 (0.49)	<0.001
A total score of psychopathological symptoms: Mean (SD)	17.39 (22.10)	10.21 (11.19)	40.22 (30.98)	<0.001
Female sex: *N* (%)	628 (48.9)	493 (50.5)	135 (44.0)	0.053
Male sex: *N* (%)	655 (51.1)	483 (49.5)	172 (56.0)	

Abbreviation: SD, standard deviation.

### Severe psychopathological symptoms and the need for psychiatric care

**Figure 1.**
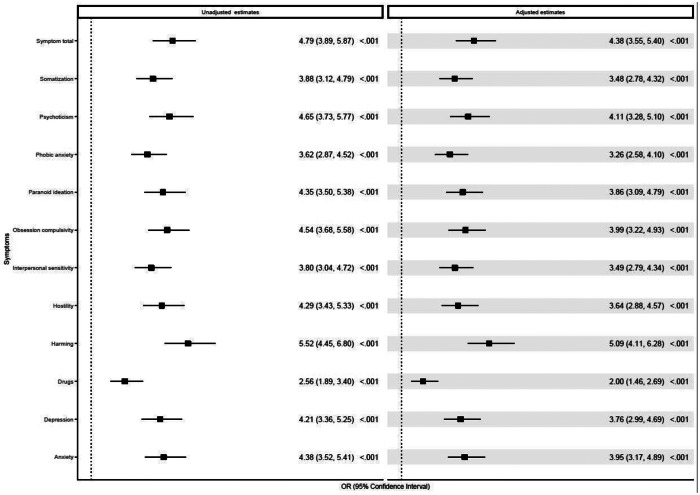
Logistic regression modeling: severe psychopathological symptoms and the need for psychiatric care. OR, odds ratio. Logistic regression models were computed unadjusted (without covariates) and adjusted for age, sex, and the total intellectual assessment score.

A total of 9.67%(N=124) of adolescents were categorized as with severe psychopathological symptoms (based on the unadjusted top 10th percentile of the total symptoms score), of whom 83.87%(N=104) were categorized as in need of psychiatric care. Of those adolescents categorized without severe psychopathological symptoms (N=1159), 17.52% (N=203) were identified as in need of psychiatric care (Supplementary Table S1). Logistic regression modeling showed that adjusted severe psychopathological symptoms were associated with a 4.4-fold increase in the need for psychiatric care compared to non-severe psychopathological symptoms (95% CI:3.55, 5.40, p<0.001; unadjusted HR=4.79; 95% CI: 3.89, 5.87, p<0.001). Results were similar for all (adjusted and unadjusted) psychopathology subscale scores (Figure 1) and remained statistically significant (*p* < 0.05) across all sensitivity analyses (Supplementary Tables S2–S5).

### The utility of severe psychopathological symptoms for clinical prediction of need for psychiatric care

Severe psychopathological symptoms had an adjusted classification accuracy of 83% (95% CI: 81%, 85%), sensitivity of 70% (95% CI: 63%, 76%), specificity of 86% (95% CI: 84%, 88%), a positive predictive value of 0.53 (95% CI: 0.48, 0.59), a negative predictive value of 0.93 (95% CI: 0.91, 0.94) and a number needed to diagnose of 1.78 (95% CI: 1.56, 2.10). Analyses of the eleven symptom subscales point to an accuracy rate lower than 84% (Table 4). Sensitivity analyses demonstrated that the performance of severe psychopathological symptoms for clinical prediction of need for psychiatric care was generally insufficient for participants with a low intellectual assessment score, males, females, and with a symptom severity categorization threshold of 20% (Supplementary Tables S6–S9).

**Table 4.** Adjusted classification indices estimating the utility of severe psychopathological symptoms for clinical prediction of need for psychiatric care.

	TP	FP	FN	TN	Accuracy (95% CI)	Sensitivity (95% CI)	Specificity (95% CI)	PPV (95% CI)	NPV (95% CI)	NND (95% CI)
Anxiety	108	199	43	933	0.81 (0.79, 0.83)	0.72 (0.64, 0.79)	0.82 (0.80, 0.85)	0.35 (0.30, 0.41)	0.96 (0.94, 0.97)	1.85 (1.58, 2.29)
Depression	116	191	50	926	0.81 (0.79, 0.83)	0.70 (0.62, 0.77)	0.83 (0.81, 0.85)	0.38 (0.32, 0.43)	0.95 (0.93, 0.96)	1.89 (1.62, 2.33)
Drug use	24	283	15	961	0.77 (0.74, 0.79)	0.62 (0.45, 0.77)	0.77 (0.75, 0.80)	0.08 (0.05, 0.11)	0.98 (0.97, 0.99)	2.58 (1.78, 5.14)
Self-harming behaviors	94	213	1	975	0.83 (0.81, 0.85)	0.99 (0.94, 1.00)	0.82 (0.80, 0.84)	0.31 (0.26, 0.36)	1.00 (0.99, 1.00)	1.23 (1.19, 1.35)
Hostility	122	185	53	923	0.81 (0.79, 0.84)	0.70 (0.62, 0.76)	0.83 (0.81, 0.85)	0.40 (0.34, 0.45)	0.95 (0.93, 0.96)	1.89 (1.62, 2.31)
Interpersonal sensitivity	85	222	47	929	0.79 (0.77, 0.81)	0.64 (0.56, 0.73)	0.81 (0.78, 0.83)	0.28 (0.23, 0.33)	0.95 (0.94, 0.96)	2.22 (1.80, 2.95)
Obsession–compulsion	134	173	56	920	0.82 (0.80, 0.84)	0.71 (0.63, 0.77)	0.84 (0.82, 0.86)	0.44 (0.38, 0.49)	0.94 (0.93, 0.96)	1.83 (1.58, 2.20)
Paranoid ideation	131	176	57	919	0.82 (0.80, 0.84)	0.70 (0.63, 0.76)	0.84 (0.82, 0.86)	0.43 (0.37, 0.48)	0.94 (0.92, 0.96)	1.87 (1.61, 2.26)
Phobic anxiety	94	213	53	923	0.79 (0.77, 0.81)	0.64 (0.56, 0.72)	0.81 (0.79, 0.83)	0.31 (0.26, 0.36)	0.95 (0.93, 0.96)	2.21 (1.81, 2.90)
Psychoticism	142	165	57	919	0.83 (0.81, 0.85)	0.71 (0.65, 0.78)	0.85 (0.82, 0.87)	0.46 (0.41, 0.52)	0.94 (0.92, 0.96)	1.78 (1.55, 2.13)
Somatization	103	204	48	928	0.80 (0.78, 0.82)	0.68 (0.60, 0.76)	0.82 (0.80, 0.84)	0.34 (0.28, 0.39)	0.95 (0.94, 0.96)	1.99 (1.67, 2.51)
A total score of psychopathological symptoms	164	143	71	905	0.83 (0.81, 0.85)	0.70 (0.63, 0.76)	0.86 (0.84, 0.88)	0.53 (0.48, 0.59)	0.93 (0.91, 0.94)	1.78 (1.56, 2.10)

*Note:* The classification indices were adjusted for age, sex, and the total intellectual assessment score.

Abbreviations: FN, false negative; FP, false positive; NND, number needed to diagnose; NPV, negative predictive values; PPV, positive predictive values; TN, true negative; TP, true positive.

## Discussion

Based on an adolescent cohort in a unique setting characterized by equal and unrestricted access to mental health professionals, we aimed to capture adolescents in need of psychiatric care with psychopathological symptoms. The results showed strong statistically significant (*p* < 0.001) associations between severe psychopathological symptoms and the need for psychiatric care. However, the clinical utility of self-reported psychopathological symptoms for classification purposes was not supported. These results were identified in a large population-based cohort of adolescents and found robust across different demographic subpopulations.

Severe psychopathological symptoms were associated with the need for psychiatric care, which is consistent with prior observational studies of children (e.g., [34]). However, psychopathological symptoms did not adequately capture adolescents in need of psychiatric care. Specifically, two thirds (66.12%) of all adolescents in need of psychiatric care were classified as without severe psychopathological symptoms, and roughly one in six (16.13%) of those classified with severe psychopathological symptoms was not classified as in need of psychiatric care. Similar to prior prodromal research (e.g., [35]), our study data showed that self-reported severe psychopathological symptoms were unreliable for classification purposes. Self-reported severe psychopathological symptoms, although accounting for a fair proportion of treatment seeking, would perhaps be better useful alongside other variables (e.g., early life risk factors; [36]) to identify adolescents at-risk.

## Limitations

The current study has some limitations. First, this study is based on traditional psychiatric taxonomies that are restricted in capturing the complexities of emerging mental disorders [37]. While new approaches are needed to generate clinical definitions that both recognize the fluid developmental course of mental illnesses and are suitable for implementation, these taxonomies still dominate the international classification systems [37]. Second, the current study identified clinically relevant psychopathological symptoms with the BSI [17]. Hence we cannot ascertain the extent to which the current results would replicate using other measures, such as the Child Behavior Checklist [38], the Strengths and Difficulties Questionnaire [39], or the Development and Well-Being Assessment [40]. Third, our conclusions with regards to psychopathological symptoms are restricted to self-reports. Past research has shown that the prevalences of self-reported psychopathological symptoms in children and adolescents is much higher than those reported by external evaluators [41]. Had clinical assessments been available, different conclusions may have emerged. Fourth, the prevalence of symptomatology ascertained by the symptom screener is based on the last month. Perhaps screening for a lifetime history would have increased the classification rates, although it may superfluously increase the rate by introducing more memory recall biases [42]. Fifth, psychopathological symptoms were categorized as severe if they were in the top 10th percentile of symptoms (otherwise not severe), leaving our study vulnerable to the limitations of dichotomization (e.g., loss of information [43]). However, in clinical and observational studies, cut-offs are widely used to ascertain severity [44], and to compute widely understood clinically values like the Number Needed to Treat [45]. Also, the current study analysis showed consistent results across cut-off thresholds, indicating that these are quite robust thresholds worthy of future research. Sixth, the sample size prohibited the scrutinization of adolescents with a specific diagnosis due to insufficient statistical power. We, therefore, analyzed a sample of participants with a vast array of reported symptoms and accounted for age, sex, and intellectual ability. Seventh, we did not test for multiple comparisons within the BSI subscales because it may lead to errors of interpretation [46]. Eighth, our study did not include hold-out data (i.e., a portion of the data that is not included in the analytic data set for validating research models). Given the combination of relatively rare-exposure (severe psychopathological symptoms) and rare-outcome (referral to a mental health professional) in our data, the primary model approach was chosen without a hold-out mechanism to ensure a reasonable level of statistical power. Future research with a prospective cross-validation sampling design is warranted to examine the role of psychopathological symptoms as a proper clinical prediction tool.

## Summary

The current study is the first prospective study of adolescents with unrestricted professional mental health care access that accounts for intellectual abilities and examines classification indices. The results show consistent, strong, and statistically significant associations between severe psychopathological symptoms and the need for psychiatric care. However, in our study data, self-reported severe psychopathological symptoms were unreliable for classification purposes required to implement mental health care policies. Self-reported severe psychopathological symptoms, although accounting for a fair proportion of treatment seeking, would perhaps be better useful alongside other variables (e.g., poverty and social disadvantage [5]) rather than in isolation.

## Data Availability

The data that support the findings of this study are not publicly available. Data may be requested from J.G. and S.F., and pending approval from the Department of Behavioral Sciences, Israel Defense Forces, Israel.

## Supplementary material

For supplementary material accompanying this paper visit https://doi.org/10.1192/j.eurpsy.2021.2251. click here to view supplementary material
