# Supplementary material for: Capturing adolescents in need of psychiatric care with psychopathological symptoms: A population-based cohort study
Source: Eur Psychiatry. 2021 Nov 29;64(1):e76. doi: 10.1192/j.eurpsy.2021.2251 (PMC8727710; doi:10.1192/j.eurpsy.2021.2251)
Supplement: Supplementary file 1 [file S0924933821022513sup001.docx]

**Supplementary material:**

**Capturing Adolescents in Need of Psychiatric Care** **with Psychopathological Symptoms:**

**A Population-Based Cohort Study**

Contents

[sFigure 1: Study flow diagram 2](#_Toc89289350)

[sTable 1: Unadjusted classification indices estimating the utility of severe psychopathological symptoms for clinical prediction of need for psychiatric care for the total sample 3](#_Toc89289351)

[sTable 2: Severe psychopathological symptoms and the need for psychiatric care in participants with a low intellectual assessment score 4](#_Toc89289352)

[sTable 3: Severe psychopathological symptoms and the need for psychiatric care in males 5](#_Toc89289353)

[sTable 4: Severe psychopathological symptoms and the need for psychiatric care in females 6](#_Toc89289354)

[sTable 5: Severe psychopathological symptoms and the need for psychiatric care in participants with a symptom severity categorization threshold of 20% 7](#_Toc89289355)

[sTable 6: Classification indices estimating the utility of severe psychopathological symptoms for clinical prediction of need for psychiatric care in participants with a low intellectual assessment score 8](#_Toc89289356)

[sTable 7: Classification indices estimating the utility of severe psychopathological symptoms for clinical prediction of need for psychiatric care in males 9](#_Toc89289357)

[sTable 8: Classification indices estimating the utility of severe psychopathological symptoms for clinical prediction of need for psychiatric care in females 10](#_Toc89289358)

[sTable 9: Classification indices estimating the utility of severe psychopathological symptoms for clinical prediction of need for psychiatric care in participants with a symptom severity categorization threshold of 20% 11](#_Toc89289359)

# sFigure 1: Study flow diagram


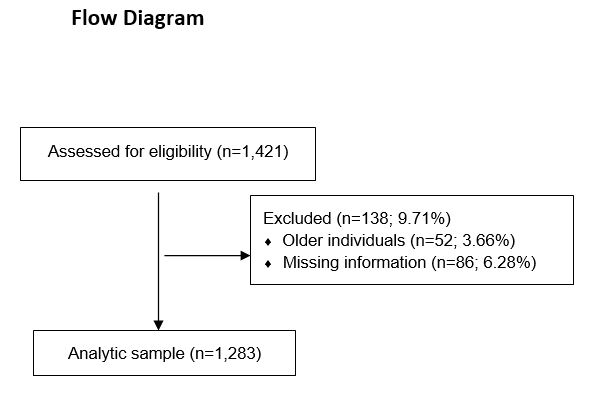


# sTable 1: Unadjusted classification indices estimating the utility of severe psychopathological symptoms for clinical prediction of need for psychiatric care for the total sample

|  | TP | FN | FP | TN | Accuracy (95% CI) | Sensitivity  (95% CI) | Specificity  (95% CI) | PPV  (95% CI) | NPV  (95% CI) | NND  (95% CI) |
| --- | --- | --- | --- | --- | --- | --- | --- | --- | --- | --- |
| Anxiety | 90 | 21 | 217 | 955 | 0.81 (0.79, 0.84) | 0.81 (0.73, 0.88) | 0.81 (0.79, 0.84) | 0.29 (0.24, 0.35) | 0.98 (0.97, 0.99) | 1.60 (1.40, 1.93) |
| Depression | 80 | 19 | 227 | 957 | 0.81 (0.79, 0.83) | 0.81 (0.72, 0.88) | 0.81 (0.78, 0.83) | 0.26 (0.21, 0.31) | 0.98 (0.97, 0.99) | 1.62 (1.41, 1.99) |
| Drug use | 39 | 30 | 268 | 946 | 0.77 (0.74, 0.79) | 0.57 (0.44, 0.68) | 0.78 (0.75, 0.80) | 0.13 (0.09, 0.17) | 0.97 (0.96, 0.98) | 2.90 (2.06, 5.12) |
| Self-harming behaviors | 94 | 1 | 213 | 975 | 0.83 (0.81, 0.85) | 0.99 (0.94, 1.00) | 0.82 (0.80, 0.84) | 0.31 (0.26, 0.36) | 1.00 (0.99, 1.00) | 1.23 (1.19, 1.35) |
| Hostility | 83 | 19 | 224 | 957 | 0.81 (0.79, 0.83) | 0.81 (0.72, 0.88) | 0.81 (0.79, 0.83) | 0.27 (0.22, 0.32) | 0.98 (0.97, 0.99) | 1.60 (1.40, 1.96) |
| Interpersonal sensitivity | 83 | 31 | 224 | 945 | 0.80 (0.78, 0.82) | 0.73 (0.64, 0.81) | 0.81 (0.78, 0.83) | 0.27 (0.22, 0.32) | 0.97 (0.96, 0.98) | 1.86 (1.57, 2.37) |
| Obsession compulsion | 101 | 24 | 206 | 952 | 0.82 (0.80, 0.84) | 0.81 (0.73, 0.87) | 0.82 (0.80, 0.84) | 0.33 (0.28, 0.38) | 0.98 (0.96, 0.98) | 1.59 (1.40, 1.90) |
| Paranoid ideation | 89 | 21 | 218 | 955 | 0.81 (0.79, 0.83) | 0.81 (0.72, 0.88) | 0.81 (0.79, 0.84) | 0.29 (0.24, 0.34) | 0.98 (0.97, 0.99) | 1.60 (1.40, 1.95) |
| Phobic anxiety | 76 | 31 | 231 | 945 | 0.80 (0.77, 0.82) | 0.71 (0.61, 0.79) | 0.80 (0.78, 0.83) | 0.25 (0.20, 0.30) | 0.97 (0.96, 0.98) | 1.95 (1.61, 2.54) |
| Psychoticism | 86 | 13 | 221 | 963 | 0.82 (0.80, 0.84) | 0.87 (0.79, 0.93) | 0.81 (0.79, 0.84) | 0.28 (0.23, 0.33) | 0.99 (0.98, 0.99) | 1.47 (1.31, 1.74) |
| Somatization | 90 | 34 | 217 | 942 | 0.80 (0.78, 0.83) | 0.73 (0.64, 0.80) | 0.81 (0.79, 0.83) | 0.29 (0.24, 0.35) | 0.97 (0.95, 0.98) | 1.86 (1.57, 2.34) |
| A total score of psychopathological symptoms | 104 | 20 | 203 | 956 | 0.83 (0.80, 0.85) | 0.84 (0.76, 0.90) | 0.82 (0.80, 0.85) | 0.34 (0.29, 0.39) | 0.98 (0.97, 0.99) | 1.51 (1.34, 1.77) |

Note. Abbreviations: TP - true positive; FP - false positive; FN - false negative; TN - true negative; PPV - positive predictive values; NPV - negative predictive values; NND - number needed to diagnose; CI - confidence intervals

# sTable 2: Severe psychopathological symptoms and the need for psychiatric care in participants with a low intellectual assessment score

|  | Estimate | Std. error | Statistic | P value | 95% CI low | 95% CI high |
| --- | --- | --- | --- | --- | --- | --- |
| Anxiety | 2.91 | 0.21 | 5.14 | <0.001 | 1.92 | 4.36 |
| Depression | 3.21 | 0.21 | 5.49 | <0.001 | 2.10 | 4.85 |
| Drug use | 2.31 | 0.25 | 3.40 | <0.001 | 1.40 | 3.70 |
| Self-harming behaviors | 3.46 | 0.22 | 5.77 | <0.001 | 2.25 | 5.24 |
| Hostility | 3.65 | 0.21 | 6.12 | <0.001 | 2.41 | 5.53 |
| Interpersonal sensitivity | 2.72 | 0.21 | 4.84 | <0.001 | 1.80 | 4.07 |
| Obsession compulsion | 3.68 | 0.21 | 6.24 | <0.001 | 2.44 | 5.53 |
| Paranoid ideation | 2.86 | 0.21 | 5.11 | <0.001 | 1.90 | 4.26 |
| Phobic anxiety | 2.73 | 0.21 | 4.74 | <0.001 | 1.79 | 4.10 |
| Psychoticism | 3.31 | 0.20 | 5.84 | <0.001 | 2.20 | 4.92 |
| Somatization | 3.04 | 0.20 | 5.49 | <0.001 | 2.04 | 4.52 |
| A total score of psychopathological symptoms | 3.40 | 0.20 | 6.05 | <0.001 | 2.28 | 5.05 |

Note. Abbreviations: Std. error - standard error; CI - confidence interval

# sTable 3: Severe psychopathological symptoms and the need for psychiatric care in males

|  | Estimate | Std. error | Statistic | P value | 95% CI low | 95% CI high |
| --- | --- | --- | --- | --- | --- | --- |
| Anxiety | 3.98 | 0.15 | 9.08 | <0.001 | 2.94 | 5.34 |
| Depression | 3.43 | 0.15 | 8.01 | <0.001 | 2.52 | 4.61 |
| Drug use | 1.97 | 0.18 | 3.74 | <0.001 | 1.36 | 2.78 |
| Self-harming behaviors | 4.84 | 0.14 | 10.98 | <0.001 | 3.63 | 6.38 |
| Hostility | 3.59 | 0.15 | 8.25 | <0.001 | 2.63 | 4.83 |
| Interpersonal sensitivity | 3.22 | 0.16 | 7.29 | <0.001 | 2.33 | 4.37 |
| Obsession compulsion | 3.84 | 0.15 | 9.23 | <0.001 | 2.88 | 5.10 |
| Paranoid ideation | 3.53 | 0.15 | 8.40 | <0.001 | 2.61 | 4.72 |
| Phobic anxiety | 3.38 | 0.16 | 7.43 | <0.001 | 2.43 | 4.63 |
| Psychoticism | 3.73 | 0.15 | 8.92 | <0.001 | 2.78 | 4.95 |
| Somatization | 3.32 | 0.16 | 7.74 | <0.001 | 2.44 | 4.48 |
| A total score of psychopathological symptoms | 3.89 | 0.15 | 9.05 | <0.001 | 2.88 | 5.19 |

Note. Abbreviations: Std. error - standard error; CI - confidence interval

# sTable 4: Severe psychopathological symptoms and the need for psychiatric care in females

|  | Estimate | Std. error | Statistic | P value | 95% CI low | 95% CI high |
| --- | --- | --- | --- | --- | --- | --- |
| Anxiety | 3.91 | 0.16 | 8.32 | <0.001 | 2.82 | 5.37 |
| Depression | 4.24 | 0.17 | 8.39 | <0.001 | 3.00 | 5.90 |
| Drug use | 2.17 | 0.30 | 2.62 | 0.01 | 1.16 | 3.73 |
| Self-harming behaviors | 5.60 | 0.16 | 10.53 | <0.001 | 4.03 | 7.67 |
| Hostility | 3.80 | 0.18 | 7.42 | <0.001 | 2.64 | 5.36 |
| Interpersonal sensitivity | 3.75 | 0.16 | 8.16 | <0.001 | 2.71 | 5.13 |
| Obsession compulsion | 4.24 | 0.16 | 8.78 | <0.001 | 3.05 | 5.83 |
| Paranoid ideation | 4.21 | 0.17 | 8.40 | <0.001 | 2.99 | 5.86 |
| Phobic anxiety | 3.06 | 0.17 | 6.48 | <0.001 | 2.17 | 4.27 |
| Psychoticism | 4.58 | 0.17 | 8.77 | <0.001 | 3.23 | 6.39 |
| Somatization | 3.58 | 0.16 | 7.76 | <0.001 | 2.58 | 4.93 |
| A total score of psychopathological symptoms | 4.93 | 0.16 | 10.13 | <0.001 | 3.61 | 6.71 |

Note. Abbreviations: Std. error - standard error; CI - confidence interval

# sTable 5: Severe psychopathological symptoms and the need for psychiatric care in participants with a symptom severity categorization threshold of 20%

|  | Estimate | Std. error | Statistic | P value | 95% CI low | 95% CI high |
| --- | --- | --- | --- | --- | --- | --- |
| Anxiety | 3.69 | 0.10 | 12.68 | <0.001 | 3.01 | 4.51 |
| Depression | 3.91 | 0.10 | 13.56 | <0.001 | 3.21 | 4.76 |
| Drug use | 2.00 | 0.16 | 4.45 | <0.001 | 1.46 | 2.69 |
| Self-harming behaviors | 5.10 | 0.11 | 15.04 | <0.001 | 4.11 | 6.28 |
| Hostility | 3.80 | 0.11 | 12.70 | <0.001 | 3.09 | 4.67 |
| Interpersonal sensitivity | 3.46 | 0.10 | 12.45 | <0.001 | 2.85 | 4.21 |
| Obsession compulsion | 4.06 | 0.10 | 13.75 | <0.001 | 3.33 | 4.96 |
| Paranoid ideation | 3.92 | 0.10 | 13.40 | <0.001 | 3.21 | 4.78 |
| Phobic anxiety | 3.30 | 0.10 | 11.50 | <0.001 | 2.69 | 4.04 |
| Psychoticism | 4.43 | 0.10 | 14.79 | <0.001 | 3.64 | 5.40 |
| Somatization | 3.57 | 0.10 | 12.40 | <0.001 | 2.92 | 4.36 |
| A total score of psychopathological symptoms | 4.81 | 0.10 | 15.23 | <0.001 | 3.94 | 5.90 |

Note. Abbreviations: Std. error - standard error; CI - confidence interval

# sTable 6: Classification indices estimating the utility of severe psychopathological symptoms for clinical prediction of need for psychiatric care in participants with a low intellectual assessment score

|  | TP | FN | FP | TN | Accuracy (95% CI) | Sensitivity  (95% CI) | Specificity  (95% CI) | PPV  (95% CI) | NPV  (95% CI) | NND  (95% CI) |
| --- | --- | --- | --- | --- | --- | --- | --- | --- | --- | --- |
| Anxiety | 31 | 31 | 9 | 89 | 0.75 (0.68, 0.81) | 0.78 (0.62, 0.89) | 0.74 (0.65, 0.82) | 0.50 (0.37, 0.63) | 0.91 (0.83, 0.96) | 1.94 (1.41, 3.71) |
| Depression | 35 | 27 | 11 | 87 | 0.76 (0.69, 0.83) | 0.76 (0.61, 0.87) | 0.76 (0.67, 0.84) | 0.56 (0.43, 0.69) | 0.89 (0.81, 0.94) | 1.91 (1.40, 3.49) |
| Drug use | 17 | 45 | 3 | 95 | 0.70 (0.62, 0.77) | 0.85 (0.62, 0.97) | 0.68 (0.59, 0.75) | 0.27 (0.17, 0.40) | 0.97 (0.91, 0.99) | 1.89 (1.38, 4.64) |
| Self-harming behaviors | 26 | 36 | 5 | 93 | 0.74 (0.67, 0.81) | 0.84 (0.66, 0.95) | 0.72 (0.64, 0.80) | 0.42 (0.30, 0.55) | 0.95 (0.88, 0.98) | 1.79 (1.35, 3.36) |
| Hostility | 38 | 24 | 11 | 87 | 0.78 (0.71, 0.84) | 0.78 (0.63, 0.88) | 0.78 (0.70, 0.86) | 0.61 (0.48, 0.73) | 0.89 (0.81, 0.94) | 1.79 (1.35, 3.04) |
| Interpersonal sensitivity | 29 | 33 | 8 | 90 | 0.74 (0.67, 0.81) | 0.78 (0.62, 0.90) | 0.73 (0.64, 0.81) | 0.47 (0.34, 0.60) | 0.92 (0.85, 0.96) | 1.94 (1.41, 3.81) |
| Obsession compulsion | 38 | 24 | 9 | 89 | 0.79 (0.72, 0.85) | 0.81 (0.67, 0.91) | 0.79 (0.70, 0.86) | 0.61 (0.48, 0.73) | 0.91 (0.83, 0.96) | 1.68 (1.30, 2.72) |
| Paranoid ideation | 41 | 21 | 13 | 85 | 0.79 (0.72, 0.85) | 0.76 (0.62, 0.87) | 0.80 (0.71, 0.87) | 0.66 (0.53, 0.78) | 0.87 (0.78, 0.93) | 1.78 (1.35, 2.97) |
| Phobic anxiety | 31 | 31 | 11 | 87 | 0.74 (0.66, 0.80) | 0.74 (0.58, 0.86) | 0.74 (0.65, 0.81) | 0.50 (0.37, 0.63) | 0.89 (0.81, 0.94) | 2.10 (1.48, 4.39) |
| Psychoticism | 39 | 23 | 9 | 89 | 0.80 (0.73, 0.86) | 0.81 (0.67, 0.91) | 0.79 (0.71, 0.87) | 0.63 (0.50, 0.75) | 0.91 (0.83, 0.96) | 1.65 (1.29, 2.62) |
| Somatization | 34 | 28 | 7 | 91 | 0.78 (0.71, 0.84) | 0.83 (0.68, 0.93) | 0.76 (0.68, 0.84) | 0.55 (0.42, 0.68) | 0.93 (0.86, 0.97) | 1.68 (1.31, 2.80) |
| A total score of psychopathological symptoms | 47 | 15 | 14 | 84 | 0.82 (0.75, 0.88) | 0.77 (0.65, 0.87) | 0.85 (0.76, 0.91) | 0.76 (0.63, 0.86) | 0.86 (0.77, 0.92) | 1.62 (1.28, 2.45) |

Note. Abbreviations: TP - true positive; FP - false positive; FN - false negative; TN - true negative; PPV - positive predictive values; NPV - negative predictive values; NND - number needed to diagnose; CI - confidence intervals

# sTable 7: Classification indices estimating the utility of severe psychopathological symptoms for clinical prediction of need for psychiatric care in males

|  | TP | FN | FP | TN | Accuracy (95% CI) | Sensitivity  (95% CI) | Specificity  (95% CI) | PPV  (95% CI) | NPV  (95% CI) | NND  (95% CI) |
| --- | --- | --- | --- | --- | --- | --- | --- | --- | --- | --- |
| Anxiety | 66 | 106 | 15 | 468 | 0.82 (0.78, 0.84) | 0.81 (0.71, 0.89) | 0.82 (0.78, 0.85) | 0.38 (0.31, 0.46) | 0.97 (0.95, 0.98) | 1.59 (1.35, 2.02) |
| Depression | 74 | 98 | 31 | 452 | 0.80 (0.77, 0.83) | 0.70 (0.61, 0.79) | 0.82 (0.79, 0.85) | 0.43 (0.36, 0.51) | 0.94 (0.91, 0.96) | 1.90 (1.56, 2.53) |
| Drug use | 19 | 153 | 11 | 472 | 0.75 (0.71, 0.78) | 0.63 (0.44, 0.80) | 0.76 (0.72, 0.79) | 0.11 (0.07, 0.17) | 0.98 (0.96, 0.99) | 2.57 (1.70, 6.33) |
| Self-harming behaviors | 53 | 119 | 0 | 483 | 0.82 (0.79, 0.85) | 1.00 (0.93, 1.00) | 0.80 (0.77, 0.83) | 0.31 (0.24, 0.38) | 1.00 (0.99, 1.00) | 1.25 (1.20, 1.43) |
| Hostility | 73 | 99 | 30 | 453 | 0.80 (0.77, 0.83) | 0.71 (0.61, 0.79) | 0.82 (0.79, 0.85) | 0.42 (0.35, 0.50) | 0.94 (0.91, 0.96) | 1.89 (1.55, 2.52) |
| Interpersonal sensitivity | 50 | 122 | 26 | 457 | 0.77 (0.74, 0.81) | 0.66 (0.54, 0.76) | 0.79 (0.75, 0.82) | 0.29 (0.22, 0.36) | 0.95 (0.92, 0.96) | 2.24 (1.71, 3.40) |
| Obsession compulsion | 80 | 92 | 30 | 453 | 0.81 (0.78, 0.84) | 0.73 (0.63, 0.81) | 0.83 (0.80, 0.86) | 0.47 (0.39, 0.54) | 0.94 (0.91, 0.96) | 1.79 (1.49, 2.32) |
| Paranoid ideation | 75 | 97 | 29 | 454 | 0.81 (0.78, 0.84) | 0.72 (0.62, 0.80) | 0.82 (0.79, 0.86) | 0.44 (0.36, 0.51) | 0.94 (0.92, 0.96) | 1.83 (1.52, 2.41) |
| Phobic anxiety | 59 | 113 | 30 | 453 | 0.78 (0.75, 0.81) | 0.66 (0.55, 0.76) | 0.80 (0.77, 0.83) | 0.34 (0.27, 0.42) | 0.94 (0.91, 0.96) | 2.16 (1.69, 3.13) |
| Psychoticism | 78 | 94 | 28 | 455 | 0.81 (0.78, 0.84) | 0.74 (0.64, 0.82) | 0.83 (0.79, 0.86) | 0.45 (0.38, 0.53) | 0.94 (0.92, 0.96) | 1.77 (1.48, 2.29) |
| Somatization | 66 | 106 | 26 | 457 | 0.80 (0.77, 0.83) | 0.72 (0.61, 0.81) | 0.81 (0.78, 0.84) | 0.38 (0.31, 0.46) | 0.95 (0.92, 0.96) | 1.89 (1.54, 2.56) |
| A total score of psychopathological symptoms | 95 | 77 | 31 | 452 | 0.84 (0.80, 0.86) | 0.75 (0.67, 0.83) | 0.85 (0.82, 0.88) | 0.55 (0.47, 0.63) | 0.94 (0.91, 0.96) | 1.64 (1.41, 2.04) |

Note. Abbreviations: TP - true positive; FP - false positive; FN - false negative; TN - true negative; PPV - positive predictive values; NPV - negative predictive values; NND - number needed to diagnose; CI - confidence intervals

# sTable 8: Classification indices estimating the utility of severe psychopathological symptoms for clinical prediction of need for psychiatric care in females

|  | TP | FN | FP | TN | Accuracy (95% CI) | Sensitivity  (95% CI) | Specificity  (95% CI) | PPV  (95% CI) | NPV  (95% CI) | NND  (95% CI) |
| --- | --- | --- | --- | --- | --- | --- | --- | --- | --- | --- |
| Anxiety | 45 | 90 | 29 | 464 | 0.81 (0.78, 0.84) | 0.61 (0.49, 0.72) | 0.84 (0.80, 0.87) | 0.33 (0.25, 0.42) | 0.94 (0.92, 0.96) | 2.24 (1.70, 3.43) |
| Depression | 48 | 87 | 23 | 470 | 0.82 (0.79, 0.85) | 0.68 (0.55, 0.78) | 0.84 (0.81, 0.87) | 0.36 (0.28, 0.44) | 0.95 (0.93, 0.97) | 1.92 (1.53, 2.74) |
| Drug use | 6 | 129 | 4 | 489 | 0.79 (0.75, 0.82) | 0.60 (0.26, 0.88) | 0.79 (0.76, 0.82) | 0.04 (0.02, 0.09) | 0.99 (0.98, 1.00) | 2.56 (1.43, 51.46) |
| Self-harming behaviors | 39 | 96 | 1 | 492 | 0.85 (0.81, 0.87) | 0.98 (0.87, 1.00) | 0.84 (0.80, 0.87) | 0.29 (0.21, 0.37) | 1.00 (0.99, 1.00) | 1.23 (1.16, 1.49) |
| Hostility | 52 | 83 | 26 | 467 | 0.83 (0.79, 0.86) | 0.67 (0.55, 0.77) | 0.85 (0.82, 0.88) | 0.39 (0.30, 0.47) | 0.95 (0.92, 0.97) | 1.94 (1.54, 2.72) |
| Interpersonal sensitivity | 46 | 89 | 27 | 466 | 0.82 (0.78, 0.84) | 0.63 (0.51, 0.74) | 0.84 (0.81, 0.87) | 0.34 (0.26, 0.43) | 0.95 (0.92, 0.96) | 2.13 (1.64, 3.17) |
| Obsession compulsion | 51 | 84 | 24 | 469 | 0.83 (0.80, 0.86) | 0.68 (0.56, 0.78) | 0.85 (0.82, 0.88) | 0.38 (0.30, 0.47) | 0.95 (0.93, 0.97) | 1.89 (1.51, 2.65) |
| Paranoid ideation | 52 | 83 | 27 | 466 | 0.82 (0.79, 0.85) | 0.66 (0.54, 0.76) | 0.85 (0.82, 0.88) | 0.39 (0.30, 0.47) | 0.95 (0.92, 0.96) | 1.97 (1.56, 2.79) |
| Phobic anxiety | 49 | 86 | 33 | 460 | 0.81 (0.78, 0.84) | 0.60 (0.48, 0.70) | 0.84 (0.81, 0.87) | 0.36 (0.28, 0.45) | 0.93 (0.91, 0.95) | 2.27 (1.73, 3.42) |
| Psychoticism | 64 | 71 | 29 | 464 | 0.84 (0.81, 0.87) | 0.69 (0.58, 0.78) | 0.87 (0.84, 0.89) | 0.47 (0.39, 0.56) | 0.94 (0.92, 0.96) | 1.80 (1.48, 2.38) |
| Somatization | 42 | 93 | 26 | 467 | 0.81 (0.78, 0.84) | 0.62 (0.49, 0.73) | 0.83 (0.80, 0.86) | 0.31 (0.23, 0.40) | 0.95 (0.92, 0.97) | 2.21 (1.68, 3.42) |
| A total score of psychopathological symptoms | 57 | 78 | 31 | 462 | 0.83 (0.79, 0.86) | 0.65 (0.54, 0.75) | 0.86 (0.82, 0.88) | 0.42 (0.34, 0.51) | 0.94 (0.91, 0.96) | 1.99 (1.59, 2.77) |

Note. Abbreviations: TP - true positive; FP - false positive; FN - false negative; TN - true negative; PPV - positive predictive values; NPV - negative predictive values; NND - number needed to diagnose; CI - confidence intervals

# sTable 9: Classification indices estimating the utility of severe psychopathological symptoms for clinical prediction of need for psychiatric care in participants with a symptom severity categorization threshold of 20%

|  | TP | FN | FP | TN | Accuracy (95% CI) | Sensitivity  (95% CI) | Specificity  (95% CI) | PPV  (95% CI) | NPV  (95% CI) | NND  (95% CI) |
| --- | --- | --- | --- | --- | --- | --- | --- | --- | --- | --- |
| Anxiety | 108 | 199 | 43 | 933 | 0.81 (0.79, 0.83) | 0.72 (0.64, 0.79) | 0.82 (0.80, 0.85) | 0.35 (0.30, 0.41) | 0.96 (0.94, 0.97) | 1.85 (1.58, 2.29) |
| Depression | 116 | 191 | 50 | 926 | 0.81 (0.79, 0.83) | 0.70 (0.62, 0.77) | 0.83 (0.81, 0.85) | 0.38 (0.32, 0.43) | 0.95 (0.93, 0.96) | 1.89 (1.62, 2.33) |
| Drug use | 24 | 283 | 15 | 961 | 0.77 (0.74, 0.79) | 0.62 (0.45, 0.77) | 0.77 (0.75, 0.80) | 0.08 (0.05, 0.11) | 0.98 (0.97, 0.99) | 2.58 (1.78, 5.14) |
| Self-harming behaviors | 94 | 213 | 1 | 975 | 0.83 (0.81, 0.85) | 0.99 (0.94, 1.00) | 0.82 (0.80, 0.84) | 0.31 (0.26, 0.36) | 1.00 (0.99, 1.00) | 1.23 (1.19, 1.35) |
| Hostility | 122 | 185 | 53 | 923 | 0.81 (0.79, 0.84) | 0.70 (0.62, 0.76) | 0.83 (0.81, 0.85) | 0.40 (0.34, 0.45) | 0.95 (0.93, 0.96) | 1.89 (1.62, 2.31) |
| Interpersonal sensitivity | 85 | 222 | 47 | 929 | 0.79 (0.77, 0.81) | 0.64 (0.56, 0.73) | 0.81 (0.78, 0.83) | 0.28 (0.23, 0.33) | 0.95 (0.94, 0.96) | 2.22 (1.80, 2.95) |
| Obsession compulsion | 134 | 173 | 56 | 920 | 0.82 (0.80, 0.84) | 0.71 (0.63, 0.77) | 0.84 (0.82, 0.86) | 0.44 (0.38, 0.49) | 0.94 (0.93, 0.96) | 1.83 (1.58, 2.20) |
| Paranoid ideation | 131 | 176 | 57 | 919 | 0.82 (0.80, 0.84) | 0.70 (0.63, 0.76) | 0.84 (0.82, 0.86) | 0.43 (0.37, 0.48) | 0.94 (0.92, 0.96) | 1.87 (1.61, 2.26) |
| Phobic anxiety | 94 | 213 | 53 | 923 | 0.79 (0.77, 0.81) | 0.64 (0.56, 0.72) | 0.81 (0.79, 0.83) | 0.31 (0.26, 0.36) | 0.95 (0.93, 0.96) | 2.21 (1.81, 2.90) |
| Psychoticism | 142 | 165 | 57 | 919 | 0.83 (0.81, 0.85) | 0.71 (0.65, 0.78) | 0.85 (0.82, 0.87) | 0.46 (0.41, 0.52) | 0.94 (0.92, 0.96) | 1.78 (1.55, 2.13) |
| Somatization | 103 | 204 | 48 | 928 | 0.80 (0.78, 0.82) | 0.68 (0.60, 0.76) | 0.82 (0.80, 0.84) | 0.34 (0.28, 0.39) | 0.95 (0.94, 0.96) | 1.99 (1.67, 2.51) |
| A total score of psychopathological symptoms | 164 | 143 | 71 | 905 | 0.83 (0.81, 0.85) | 0.70 (0.63, 0.76) | 0.86 (0.84, 0.88) | 0.53 (0.48, 0.59) | 0.93 (0.91, 0.94) | 1.78 (1.56, 2.10) |

Note. Abbreviations: TP - true positive; FP - false positive; FN - false negative; TN - true negative; PPV - positive predictive values; NPV - negative predictive values; NND - number needed to diagnose; CI - confidence intervals
